# Simultaneous quantification of GABA, Glx and GSH in the neonatal human brain using magnetic resonance spectroscopy

**DOI:** 10.1016/j.neuroimage.2021.117930

**Published:** 2021-06

**Authors:** Yanez Lopez Maria, Anthony N. Price, Nicolaas A.J. Puts, Emer J. Hughes, Richard A.E. Edden, Grainne M. McAlonan, Tomoki Arichi, Enrico De Vita

**Affiliations:** a Centre for the Developing Brain, School of Biomedical Engineering and Imaging Sciences, King's College London, London, United Kingdom; b Department of Forensic and Neurodevelopmental Sciences, Sackler Institute for Translational Neurodevelopment, Institute of Psychiatry, Psychology & Neuroscience, King's College London, London, United Kingdom; c Russell H. Morgan Department of Radiology and Radiological Science, The Johns Hopkins University School of Medicine, Baltimore, MD, United States; d F. M. Kirby Research Center for Functional Brain Imaging, Kennedy Krieger Institute, Baltimore, Maryland, United States; e MRC Centre for Neurodevelopmental Disorders, King's College London, London, United Kingdom; f NIHR-Maudsley Biomedical Research, King's College London, United Kingdom; g Department of Bioengineering, Imperial College London, South Kensington Campus, London, United Kingdom; h Biomedical Engineering Department, School of Biomedical Engineering and Imaging Sciences, St Thomas’ Hospital, Westminster Bridge Road, Lambeth Wing, 3rd Floor, London SE1 7EH, United Kingdom

**Keywords:** Edited-MRS, Neonate, GABA, Glutamate, GSH

## Abstract

Balance between inhibitory and excitatory neurotransmitter systems and the protective role of the major antioxidant glutathione (GSH) are central to early healthy brain development. Disruption has been implicated in the early life pathophysiology of psychiatric disorders and neurodevelopmental conditions including Autism Spectrum Disorder.

Edited magnetic resonance spectroscopy (MRS) methods such as HERMES have great potential for providing important new non-invasive insights into these crucial processes in human infancy. In this work, we describe a systematic approach to minimise the impact of specific technical challenges inherent to acquiring MRS data in a neonatal population, including automatic segmentation, full tissue-correction and optimised GABA+ fitting and consider the minimum requirements for a robust edited-MRS acquisition. With this approach we report for the first time simultaneous GABA+, Glx (glutamate + glutamine) and GSH concentrations in the neonatal brain (*n* = 18) in two distinct regions (thalamus and anterior cingulate cortex (ACC)) using edited MRS at 3T.

The improved sensitivity provided by our method allows specific regional neurochemical differences to be identified including: significantly lower Glx and GSH ratios to total creatine in the thalamus compared to the ACC (*p* < 0.001 for both), and significantly higher GSH levels in the ACC following tissue-correction (*p* < 0.01). Furthermore, in contrast to adult GABA+ which can typically be accurately fitted with a single peak, all neonate spectra displayed a characteristic doublet GABA+ peak at 3 ppm, indicating a lower macromolecule (MM) contribution to the 3 ppm signal in neonates. Relatively high group-level variance shows the need to maximise voxel size/acquisition time in edited neonatal MRS acquisitions for robust estimation of metabolites.

Application of this method to study how these levels and balance are altered by early-life brain injury or genetic risk can provide important new knowledge about the pathophysiology underlying neurodevelopmental disorders.

## Highlights

• First simultaneous measurement of GABA+, Glx and GSH in the neonatal human brain.
• Robust metabolic estimation in neonates requires a specific quantification strategy.
• GABA+ has a doublet peak in neonates indicating lower macromolecular contributions.
• Future application can inform about pathophysiology in neurodevelopment.

## 1 Introduction

The neuronal-glial unit has a fundamental role during early brain development and brain function. Central to this is healthy functioning of the main inhibitory and excitatory neurotransmitters GABA and Glutamate, and the protective role of the major antioxidant glutathione (GSH). Together, GABA, Glutamate, and GSH are all part of a carefully regulated metabolic system, and are in constant flux (Rae, 2014). This early-life interplay is crucial for regulating synapse activity and maturation, with disruption to components of this carefully regulated system resulting in life-long effects on brain organization and function (Blankenship and Feller, 2010; McGann and Mandel, 2018). In keeping with this, GABA, Glu, and GSH systems have all been implicated in the pathophysiology of several psychiatric disorders and neurodevelopmental conditions including Autism Spectrum Disorder (Ajram et al., 2019; Canitano and Pallagrosi, 2017; Gaetz et al., 2014; Rae and Williams, 2017).

Neurometabolites including the aforementioned key neurotransmitter and antioxidant systems can be non-invasively measured in the human brain using magnetic resonance spectroscopy (MRS). Due to their relatively low concentration with resonances overlapping those of more highly concentrated metabolites (Harris et al., 2017), the recommended approach for their measurement is J-difference spectral edited MRS. This is the case for both GABA and GSH (1–2 mM) and to a lesser extent, Glu (4–5 mM) in the human 1H-MR spectrum. Importantly, these challenges are further compounded when attempting to perform studies in newborn infants due to specific technical and practical complexities inherent to working with this population. Metabolic concentrations for example, are generally expected to be lower in the neonatal brain than those found in adults (Morgan et al., 2013; Tomiyasu et al., 2017). Given that edited MRS already requires large volumes of interest and long acquisition times (on the order of 27 mL over a 10 min acquisition (Mikkelsen et al., 2018)) to obtain sufficient signal-to-noise ratio (SNR) for reliable measurements, neonatal acquisitions will require even larger volumes of interest or extended acquisition times. However, small brain sizes exacerbate difficulties with large voxel placement, as variations in tissue composition between subjects are known to be an important source of variance in GABA measurements in adults (Harris et al., 2015). Moreover, the uncooperative nature of neonates often leads to unpredictable patterns of head motion which complicate long acquisitions and requires specialist equipment and expertise if the subjects are not sedated (Mathur et al., 2008). Another consideration is that the 3 ppm GABA signal contains co-edited macromolecule (MM) contributions (hence this is often referred to as GABA+), which may vary with age. Techniques to remove contributions from MM exist but are also highly sensitive to motion (Near et al., 2011; Terpstra et al., 2002). As a minimum, the interpretation of edited-MRS (MEGA-PRESS, HERMES) should consider the MM contribution to the 3 ppm GABA+ signal, and the influence of frequency drift as a quantification limitation (Choi et al., 2020).

A handful of studies have measured GABA and Glx (combined glutamate and glutamine) in neonates using the MEGA-PRESS (Mescher et al., 1998) sequence (Basu et al., 2020; Kwon et al., 2014; Tanifuji et al., 2017; Tomiyasu et al., 2017) . However, these existing neonatal studies often used variable voxel sizes and acquisition durations not in line with consensus recommendations (Mullins et al., 2014), used sedation, did not apply tissue composition corrections or did not consider the MM contributions to the 3 ppm GABA+ signal. Therefore, existing neonatal work did not fully adhere to the QA standards set by the editing MRS community (GABA MEGA-PRESS basic guidelines (Mullins et al., 2014), more recent consensus recommendations on edited-MRS (Choi et al., 2020), or reporting standards (Peek et al., 2020). Together, this may result in compromised SNR, accuracy and ultimately, the reproducibility of the values reported. In addition, whilst prior studies have focused on single mechanisms (e.g. GABA, or GSH), no study has examined all three metabolites simultaneously, despite their importance and interplay in the critical period of early development.

Here, we describe methods to apply the recently published HERMES (Hadamard Encoding and Reconstruction of MEGA-Edited Spectroscopy) (Saleh et al., 2016) edited MRS sequence in a neonatal population. HERMES extends the capabilities of MEGA-PRESS by resolving in vivo overlapping GABA+, Glx and GSH signals simultaneously within a single experiment, without loss of SNR. Our aim was to simultaneously measure GABA+, GSH and Glx in a neonatal population for the first time, whilst addressing the need for a robust and specific approach by adhering to the same quality standards as adult population edited-MRS studies, in order to produce high-quality reproducible normative neonatal data. We also aimed to investigate the impact of scan duration on GABA+, Glx and GSH quantification (SNR, fit error and group level variance). Finally, to minimise the impact of specific technical challenges inherent to neonatal MRS, we assemble an automatic quantification pipeline including optimised GABA+ fitting and full tissue-correction based on optimised neonatal tissue segmentations.

## 2 Methods

### 2.1 Participants

In vivo imaging was performed on a Philips Achieva 3T system (Philips Healthcare, Best, the Netherlands) in the neonatal intensive care unit at St. Thomas Hospital (London, UK). Eighteen healthy neonates were recruited and scanned at term equivalent age (median gestation age at birth: 39 (29–41) weeks, median postmenstrual age at scan: 41 (range: 39–47) weeks). Neonates were excluded if there was a history of birth asphyxia, if they were known to have a congenital brain abnormality, or any clinical history of brain injury. All anatomical images acquired for this study were reported by an experienced neonatal neuroradiologist as showing normal brain appearances. Written informed parental consent was obtained prior to scanning (UK NHS REC code: *12*/*LO*/*1247*).

### 2.2 Data acquisition

Participants were scanned without sedation following feeding, using a dedicated neonatal imaging system (Hughes et al., 2017), including a 32-channel receive head coil (Rapid Biomedical GmbH, Rimpar, DE). A continuous soundtrack of recorded fMRI sequence gradient noise was used to help settle the baby and reduce the disturbing characteristic stop-start noise pattern associated with the transition between acquisition sequences. Following localizers, anatomical data were collected using T1-MPRAGE and T2-TSE, with the following parameters: TR/TE = 17/4.6 ms, TI  =  1465 ms, flip angle  =  13 °, voxel size = 0.82  ×  0.93  ×  1.0  mm for T1-MPRAGE; and TR/TE = 14,473/160 ms, voxel size = 1.14  ×  1.14  ×  2  mm, TSE factor = 16 for T2-TSE.

**Fig 1:**
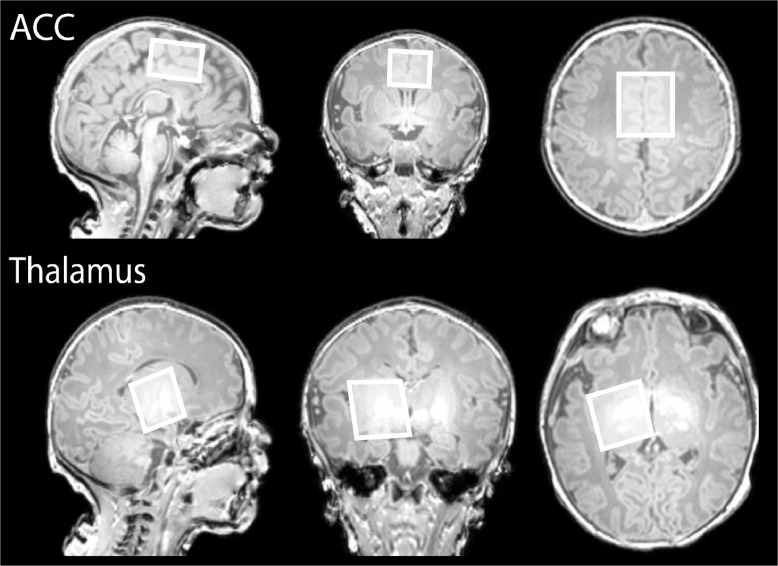
Representative voxels centred over the anterior cingulate cortex (top, 31.25×25×20 mm^3^) and the left thalamus (bottom, 25×25×25 mm^3^, neurological convention) used for the MRS acquisitions, superimposed on the T1-weighted data. The excitation frequency was set to the proton resonance of creatine (3 ppm). Neonate gestation age at birth: 39+2 weeks, postmenstrual age at scan: 39+0 weeks.

Single voxel 1H-MRS data were acquired in two different voxels of identical total volume following ‘pencil-beam’ shimming: a 31.25 × 25 × 20 mm^3^ voxel of Interest (VOI) over the anterior cingulate cortex and a 25 × 25 × 25 mm^3^ VOI over the left thalamus (see Fig 1, neurological convention). HERMES MRS was used with the following parameters (see also (Saleh et al., 2016)): TE/TR: 80/2000 ms, 2 kHz receiver bandwidth, 2048 data points, 320 averages, 90° excitation/180° refocusing pulses and 20 ms editing pulses at 1.9 ppm for GABA and 4.56 ppm for GSH, with VAPOUR water suppression. The excitation frequency was set to the proton resonance of creatine (3 ppm). Prospective frequency correction for field drift during acquisition was performed using interleaved water referencing (Edden et al., 2016).

### 2.3 Data analysis

HERMES MRS data were pre-processed and quantified using Gannet 3.1 (http://www.gabamrs.com/downloads) (Edden et al., 2014), including line broadening (3 Hz), eddy current correction and spectral registration (Near et al. 2015), with custom modifications as described in the following paragraphs. Two ACC datasets and four thalamus datasets were excluded due to incomplete acquisitions as a result of the infant waking. Following qualitative evaluation, 2 Glx datasets and 1 GSH dataset were excluded from further analysis due to poor water suppression and spectral alignment issues respectively.

GABA+, Glx and GSH levels were first quantified relative to the unsuppressed water signal and as ratios to total creatine, with fit errors estimated by weighted least squares model fitting. Two different fits of the GABA+ peak were performed, using either a single Gaussian or a double Gaussian model. A single Gaussian function has been shown to be a good approximation for GABA+ adult spectra (Edden et al., 2014; Mullins et al., 2014), whilst a double Gaussian model more closely matches the approach of basis file-based software for the 3 ppm pseudo-doublet of the GABA signal (i.e. two single Gaussian peaks in TARQUIN (Wilson et al., 2011)). Both approaches have been shown to produce identical results in adults. GSH was fitted using the default Gannet approach (Saleh et al., 2016).

Anatomical brain images were segmented into tissue types using the developing Human Connectome Project structural pipeline (Makropoulos et al., 2018), which uses age-specific neonatal brain templates and provides tissue fraction maps for grey matter (GM), white matter (WM) and CSF classifications. After co-registering the MRS voxel coordinates onto the T2 images using a modified version of GannetCoReg, the different tissue fractions in the two voxels of interest were estimated for each subject. Tissue correction was applied to all metabolic concentrations (Harris et al., 2015) with no assumed ratio between the GABA concentrations in WM and GM, as established reference values have been derived from adult populations. Published neonatal values were used for the parameters T1, T2 and water concentration of GM, WM and CSF at 3T (Williams et al., 2005). The T1 and T2 values of metabolites GABA, Glx and GSH were interpolated by multiplying adult values by the estimated water relaxation ratios of neonates to adults (T1 water neonate/adult ratio = 1.12, T2 water neonate/adult ratio = 1.44).

In order to test the dependence of concentration estimates on scan duration, all datasets were also analysed with a cumulative number of averages (NA), from NA = 80 to NA = 320, in steps of 20 NA, producing 12 spectra with increasing NA. Metabolite levels (ratios to total creatine), SNR and model fit error parameters were derived, and the coefficient of variation was calculated at group level.

### 2.4 Statistical analysis

Results are expressed as average estimated metabolic concentrations ± standard error of the mean across subjects. Unpaired t-tests were performed to investigate differences between metabolic concentrations in the two anatomical regions.

## 3 Results

HERMES MRS data was successfully quantified in a minimum of 13 of the subjects for each of the metabolites and regions studied. The relevant number of datasets included are reported in Table 1 together with a number of quantitative quality metrics (average fit error, linewidth, SNR and frequency drift).

**Table 1:** Number of datasets included in final analysis and quantitative quality metrics (Gannet). Quantitative quality metrics are GABA+/Glx/GSH fit errors; NAA linewidth; NAA SNR; GABA+/Glx/GSH SNR; and average frequency offset change ∆δ_0._

Number of datasets included in analysis	ACC	thalamus
GABA+	16	14
Glx	14	14
GSH	16	13
**Quantitative quality metrics**	**ACC**	**thalamus**
GABA+ fit error (%)	7.9 ± 0.9	6.3 ± 0.6
Glx fit error (%)	3.1 ± 0.2	3.0 ± 0.3
GSH fit error (%)	21.7 ± 4.1	27.1 ± 3.3
NAA linewidth (Hz)	9.0 ± 0.3	7.4 ± 0.4
NAA SNR	80.4 ± 6.9	97.8 ± 8.2
GABA+ SNR	6.8 ± 0.9	8.7 ± 0.9
Glx SNR	15.4 ± 1.6	18.1 ± 1.7
GSH SNR	8.9 ± 0.7	8.9 ± 0.8
∆δ_0_ (ppm)	0.032 ± 0.003	0.030 ± 0.003

**Fig 2:**
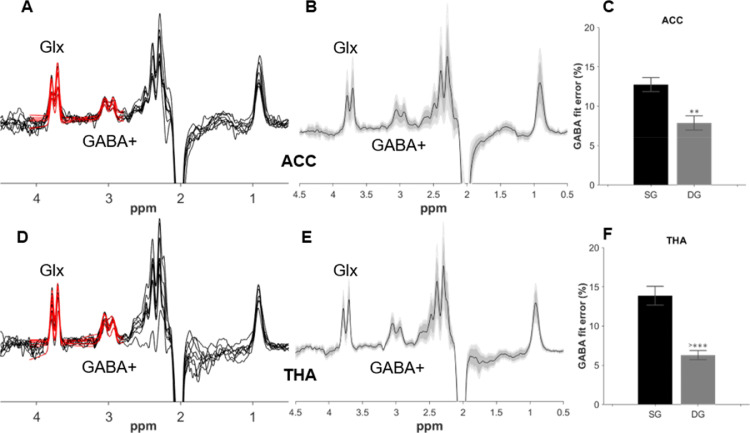
GABA+ and Glx edited spectra from all neonates from the ACC (A) and thalamus (D) are plotted in black, with double Gaussian fitted model in red. Corresponding group averages from the ACC (B) and thalamus (E) in black, with the standard deviation in dark grey and the 95% confidence interval in light grey. GABA+% fit error (C, F) showing the difference in GABA+ fitting between single Gaussian (SG) and the double Gaussian (DG) models.

**Fig 3:**
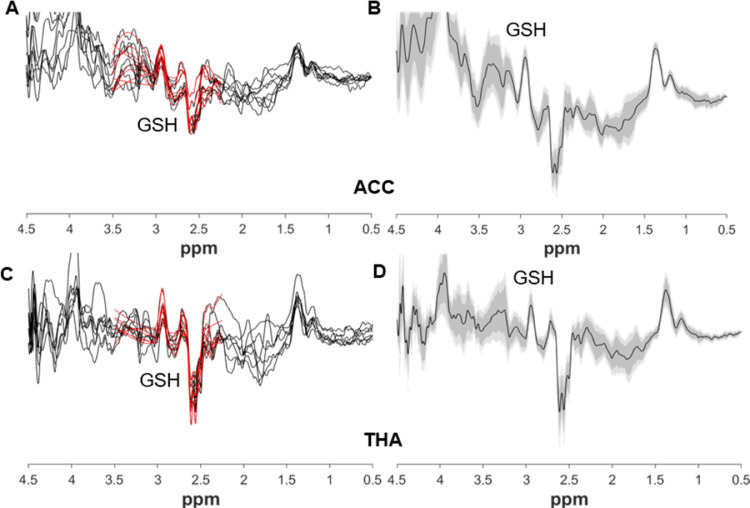
GSH-edited spectra from all neonates from the ACC (A) and thalamus (C) plotted in black, with fitted model in red. Corresponding group averages from the ACC (B) and thalamus (D) in black, with the standard deviation in dark grey and the 95% confidence interval in light grey.

All edited GABA+/Glx edited spectra, and group averages, are displayed in Fig 2, together with GABA+ fit errors. All neonates displayed a clear doublet GABA+ peak at 3 ppm; moreover, the GABA+ fit errors for the double Gaussian fitting approach were significantly lower compared with those obtained using the single Gaussian model (*p* < 0.001 for both voxels). GABA+ fitted values were higher when using the single Gaussian model (GABA+/water_ACC_ = 0.94 ± 0.07 and GABA+/water_THA_ = 1.31 ± 0.11 for the single Gaussian model, versus GABA+/water_ACC_ = 0.75 ± 0.07 and GABA+/water_THA_ = 1.03 ± 0.08 for the double Gaussian model). The GSH edited individual spectra and group averages are displayed in Fig 3.

**Fig 4:**
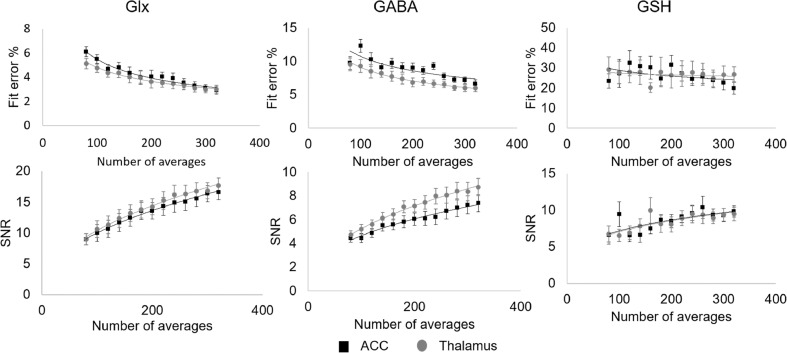
Group average SNR and fit error percentages as a function of scan duration (cumulative signal averaging) for GABA+, Glx and GSH in the ACC (black squares) and thalamus (grey circles). The fitted lines represent the square-root (for SNR) or inverse square-root (for the fit error) expected relationships. SNR increases with the square root of the number of averages for all three metabolites (R^2^ > 0.9 for GABA+ and Glx, R^2^ > 0.7 for GSH). GABA+ and Glx fit errors decrease with the square root of the number of averages (R^2^ > 0.9), with a weaker negative relationship for GSH (R^2^ ~ 0.2).

For GABA+ and Glx, SNR and fit error showed positive and negative strong relationships respectively with the square root of the number of averages (R^2^ > 0.9, see Fig 4) in both voxels, with higher GABA+ SNR and lower fit error in the thalamus. GSH SNR similarly increased with the square root of the number of averages (R^2^ > 0.7), though a much weaker negative relationship was seen for the fit error (R^2^ ~ 0.2).

**Fig 5:**
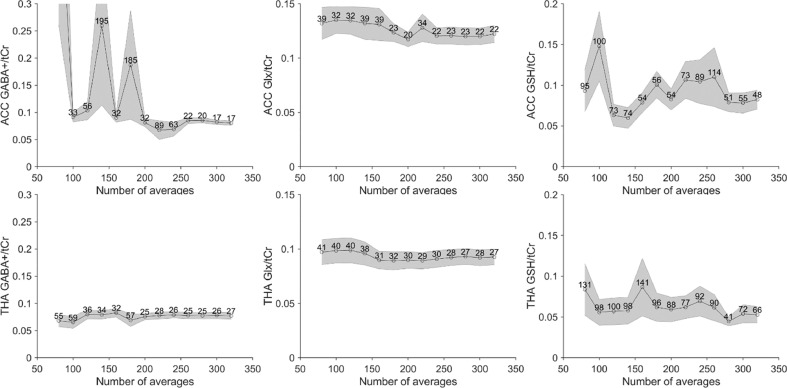
Group average GABA+/Glx/GSH ratios to total creatine as a function of scan duration (cumulative signal averaging) in the ACC (top row) and thalamus (bottom row). The labels represent the coefficient of variation as a percentage.

Estimated concentration ratios are shown in Fig 5 as a function of the cumulative number of averages, together with the group-level coefficients of variation (CV). The group-level variance of GABA+/Cr and Glx/Cr measurements stabilizes towards the maximum number of averages in the ACC and slightly fewer in the thalamus. However, this is not the case for GSH, which benefits from increasing signal averaging.

**Fig 6:**
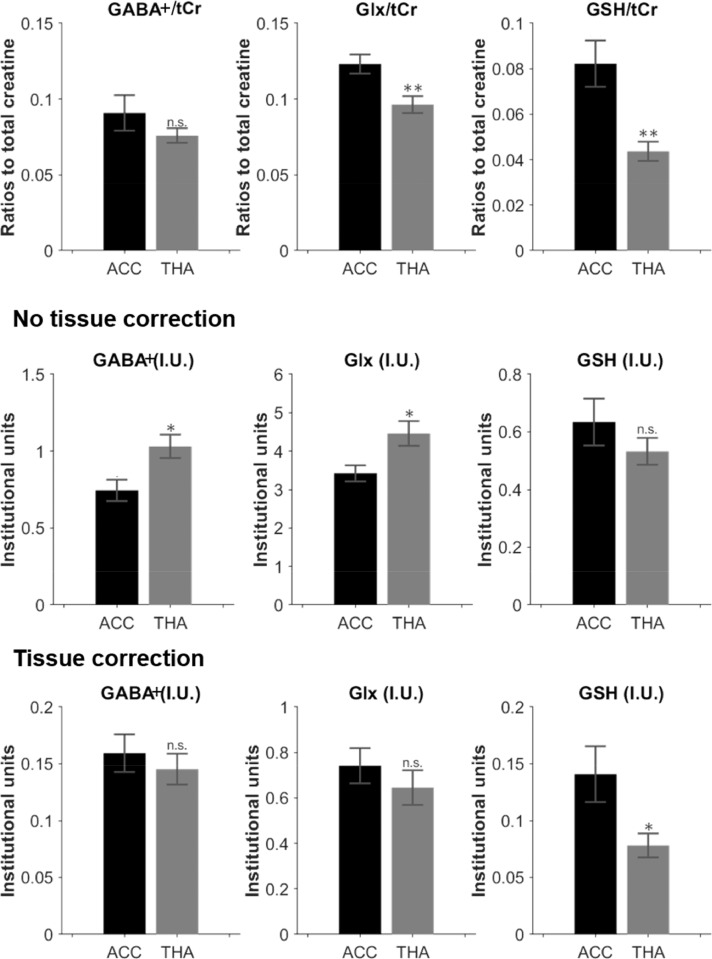
Estimated metabolic concentrations for GABA+, Glx and GSH, expressed as the ratios to total creatine (top row).Estimated metabolic concentrations for GABA+, Glx and GSH in institutional units, with the water signal used as reference before tissue correction (middle row), and after tissue correction (bottom row). *: *p*<0.01; **: *p*<0.001.

Group averaged metabolite concentration ratios, as well as individual metabolite concentrations scaled to water are shown in Fig 6, with and without tissue composition correction. Glx/tCr and GSH/tCr ratios were significantly lower in the thalamus compared with the ACC (*p* < 0.001 for both, Fig 6- top row).

**Fig 7:**
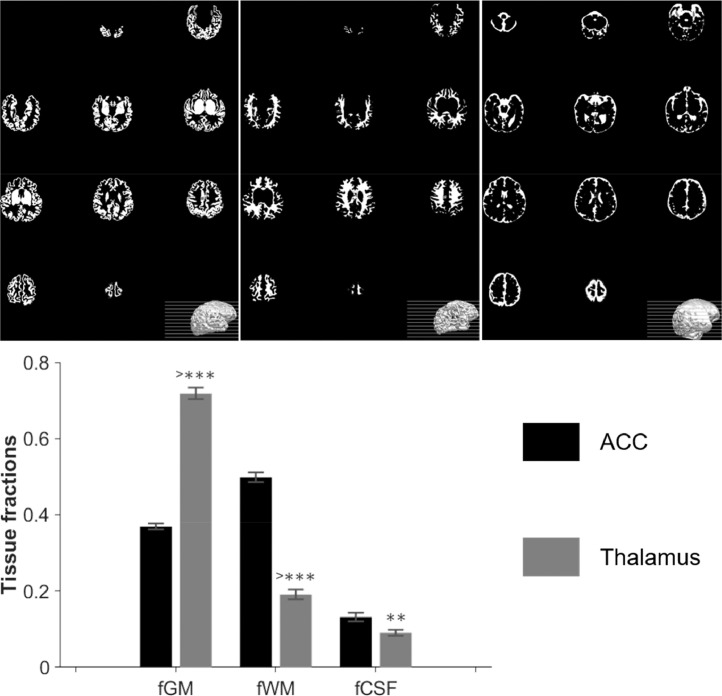
Top: Representative neonatal segmentation using the developing Human Connectome Project structural pipeline. From left to right, maps for GM, WM and CSF obtained from the T2 images. Bottom: group averaged tissue fractions in the two volumes of interest, ACC and thalamus. *: *p*<0.01; **: *p*<0.001; ***: *p*<0.0001.

Voxel tissue segmentation results are shown in Fig 7. GM tissue fraction was significantly higher in the thalamus compared with the ACC, whilst WM and CSF were lower. Before tissue correction was applied, GABA+/water and Glx/water were significantly higher in the thalamus (*p* < 0.01 for both, Fig 6- middle row), but after accounting for tissue fraction composition variations between the two voxels (Fig 6- bottom row), differences in GABA+ and Glx levels between regions were no longer significant. GSH levels were significantly higher in the ACC vs thalamus following tissue correction (*p* < 0.01).

There were no significant postmenstrual age relationships for any of the metabolite concentrations (see Supplementary Table 1).

## 4 Discussion

We report an acquisition and analysis pipeline for in vivo estimation of GABA+, Glx and GSH in the human neonatal brain using the HERMES MRS sequence. In addition to providing metabolite values in this population within two important regions of the developing brain, we also demonstrate several requirements to ensure robust metabolite estimation taking into account population-specific characteristics.

### 4.1 GABA± 3 ppm peak

All of the neonatal spectra acquired displayed a characteristic doublet GABA+ peak at 3 ppm in our study, which contrasts with the single peak frequently seen in adult in vivo measurements (Mikkelsen et al., 2017). The GABA+ signal contains contributions from GABA and MM, the latter considered to originate from the amino acid lysine (Behar and Ogino, 1993). As a result, the expected pseudo-doublet character of the 3 ppm signal is not always observed in adult spectra, due to the presence of MM contributions. Moreover, creatine subtraction artefacts can also be misinterpreted as ‘true’ doublet splittings (Evans et al., 2013; Mullins et al., 2014), but in our neonatal subjects, the doublet splitting shape was observed even in subjects with minimal drift in the frequency of the creatine peak. As a result, selecting a double Gaussian model for fitting our data significantly improved the fit and resolved underestimation of the signal.

A previous neonatal study (Tomiyasu et al., 2017) used a Lorentzian model with five variable parameters to fit bimodal GABA and MM peaks in neonates and showed lower in vivo GABA+ levels in neonates compared to children in the basal ganglia and cerebellum, attributing this to changes in GABA and not MM, based on the observation that MM was reported to be stable over time from teenage to adult subjects (Hofmann et al., 2001; Mader et al., 2002). However, the progression of MM levels cannot be necessarily extrapolated from teenagers to neonates.

The doublet nature of the 3 ppm peak reported in our work indicates that a lower MM contribution to the GABA+ 3 ppm signal is likely to be present in neonates; this contribution presumably increasing with development, and needs to be considered in the fitting process. In keeping with this, previous preclinical work has showed a 30% increase in overall macromolecular content measured with MRS during postnatal development (Tkáč et al., 2003). Neuropeptide levels are also known to increase in the first three weeks after birth (Polin et al., 2011), and l-pipecolate levels (the by-products of lysine breakdown) are much higher around birth than in adults (as large amounts of lysine are being broken down) and then decrease over time (Hallen et al., 2013). Additionally, a recent study in children aged 7–14 years has suggested that previously reported changes in GABA+ with age could have been driven by changes in MM levels (Bell et al., 2021).

### 4.2 Scan duration and voxel characteristics

Half of the previous neonatal edited-MRS studies have used extremely small voxel sizes (3.7–9.4 mL) and all have used shorter acquisition times (3.2–8.5 min). Moreover, metabolic concentrations are generally lower in the neonatal brain than in adults, and thus the use of smaller voxel sizes and/or shorter acquisition times need to be carefully evaluated. Our results have shown increased SNR in GABA+, Glx and GSH with cumulative number of averages, following an expected square-root function. We also show decreased GABA+ fit error with increased number of averages (inverse square-root dependency), as was previously described in simulated edited GABA data and in an adult population (Mikkelsen et al., 2018). In that study, negligible gains in the decrease of the group-level variance were observed after a certain number of averages (~200, voxel dependant) with subsequent recommendations for a reduction in the MRS acquisition duration. Our neonatal MRS data shows increased variability in the GABA+ group-level variance compared to the adult data, which could be explained by a combination of factors. First, to maintain anatomical specificity, a smaller voxel is required - we chose 15.625 mL relative to a typical 27 mL used in adults- and the GABA+ concentration is lower, i.e. the measured ACC neonatal GABA+ concentration is 0.16 mM versus reported 1.72 mM in adults (Flores-Ramos et al., 2019). Another factor is the effect of random neonatal motion, likely responsible for an average measured frequency offset change of ~ 0.03 ppm, which is higher than most of the adult values reported for Philips scanners benefitting from frequency correction during data acquisition (Mikkelsen et al., 2017). Therefore, we suggest that time savings in the MRS acquisition are not advisable for neonates since this would increase the group variance and thus reduce the degree of statistical power. Given the pseudo-random timing of neonatal motion, longer acquisitions are beneficial to enable data redundancy for corrupted averages. Additionally, fitting of GSH is more complicated than for GABA+, and GSH has a higher group-level variance (as illustrated in Fig 5), so additional approaches should be discussed for better GSH quantification.

Looking at the two voxels separately, fit errors are higher and SNR is lower in the ACC data compared to the thalamus for all metabolites. This is most likely due to underlying B_0_ homogeneity within and around the voxel of interest. The thalamus is in a central brain region, compared with the ACC which is closer to the skull: therefore thalamic spectra will experience reduced motion-induced spectral distortions. Accordingly, NAA linewidth, as well as the average frequency offset change during acquisition are significantly higher in the ACC datasets.

### 4.3 Tissue and relaxation correction

In previous neonatal edited-MRS work, tissue and relaxation corrections were either not performed (Basu et al., 2020; Tanifuji et al., 2017) or only partially applied. For instance, Kwon et al. used a GM-only correction, assuming that WM contains no GABA (Kwon et al., 2014), and Tomiyasu et al. included a relaxation correction for water and metabolites without any tissue correction (no segmentation (Tomiyasu et al., 2017)). We have implemented for the first time, a full correction for relaxation and tissue composition for GABA+ and GSH measurements in neonates, based on a neonate-optimised structural data segmentation pipeline (Makropoulos et al., 2018). Indeed, it is well known - and we have shown here - that without tissue correction, MRS results are heavily driven by differences in tissue content between anatomical regions.

To perform relaxation correction, literature-derived relaxation parameters of water (T1_WM_, T2_WM_, T1_GM_ and T2_GM_,) were adopted for all neonates from average population values (Williams et al., 2015). T1 and T2 of metabolites (GABA, Glx and GSH) have yet to be measured in neonates which could be the study of future work. Our current approach estimates these by interpolation of adult values and known water relaxation ratios of neonates to adults. Our resulting tissue-composition correction (including water and metabolite relaxation) improves the measurement's precision by removing contributions to inter-subject variance from tissue composition in the acquired volumes, while also improving the accuracy of the metabolite concentration estimation (Harris et al., 2015).

### 4.4 Frequency drift

B0 frequency drift during data acquisition (hardware or subject-motion related) has a significant effect on editing efficiency and co-editing contributions in edited-MRS (much more so than in conventional MRS (Andronesi et al., 2020; Choi et al., 2020). Although post-processing frequency/phase correction can improve subtraction artifacts in MEGA-PRESS/HERMES, only real-time frequency correction during acquisition can avoid signal and editing efficiency loss.

Mitigating drift/offsets is even more important in neonatal edited-MRS, due to the non-cooperative nature of the subjects. Half of the previous studies have used sedation, which can have confounding effects (Makaryus et al., 2011) on normative values, and none have used real-time frequency correction. In this work, frequency correction for field drift during acquisition was performed using interleaved water referencing (Edden et al., 2016), to reduce the effect of random neonatal motion. Ideally, prospective real-time movement correction (Andronesi et al., 2020) could be combined with the real-time frequency correction used here and this could be explored in future work.

## Conclusion

We describe a systemic approach for acquiring robust and reproducible edited MRS data from the challenging neonatal population. This has allowed us to report the first simultaneous GABA+, Glx and GSH measurements in a cohort of healthy human neonates at 3T and demonstrates that HERMES can be used to measure significant regional differences between the thalamus and anterior cingulate cortex in this population, but a specific fitting and quantification strategy is crucial to obtain high-quality reproducible normative data. The presence of a neonatal GABA+ doublet suggests a lower macromolecular contamination than in adults in the 3 ppm resonance, and future studies of age‐related MM contribution rates are required to further improve the accuracy of measurement of GABA+ levels in neonates. Applications of this method to study how these metabolite levels and their balance are altered by early-life brain injury or genetic risk can provide important new knowledge about the pathophysiology underlying neurodevelopmental disorders.

## CRediT authorship contribution statement

**Yanez Lopez Maria:** Conceptualization, Methodology, Software, Validation, Formal analysis, Investigation, Data curtion, Writing – original draft, Writing – review & editing, Visualization. **Anthony N. Price:** Software, Investigation, Resources. **Nicolaas A.J. Puts:** Conceptualization, Methodology, Software, Writing – review & editing, Visualization, Supervision. **Emer J. Hughes:** Investigation, Resources. **Richard A.E. Edden:** Software, Resources. **Grainne M. McAlonan:** Conceptualization, Writing – review & editing, Supervision, Funding acquisition. **Tomoki Arichi:** Conceptualization, Methodology, Validation, Formal analysis, Investigation, Resources, Data curtion, Writing – original draft, Writing – review & editing, Supervision, Project administration, Funding acquisition. **Enrico De Vita:** Conceptualization, Methodology, Software, Validation, Investigation, Resources, Data curtion, Writing – original draft, Writing – review & editing, Supervision, Project administration, Funding acquisition.

## Declarations of Competing Interest

None.

## Appendix Supplementary materials

Image, application 1
