# Supplementary material for: Simultaneous quantification of GABA, Glx and GSH in the neonatal human brain using magnetic resonance spectroscopy
Source: Neuroimage. 2021 Jun;233:117930. doi: 10.1016/j.neuroimage.2021.117930 (PMC8204265; doi:10.1016/j.neuroimage.2021.117930)
Supplement: Supplementary file 1 [file mmc1.docx]

*Supplementary table 1. Postmenstrual age correlations with metabolic concentrations. Analysis performed using software InVivoStat.*

| **Categorisation factor** | **First variable** |  | **Second variable** | **Correlation Coefficient** | **Test statistic** | **p-value** |
| --- | --- | --- | --- | --- | --- | --- |
| ACC | Postmenstrual age | vs. | GABA_ConcCr | -0.008 | -0.031 | 0.9758 |
| ACC | Postmenstrual age | vs. | Glx_ConcCr | -0.437 | -1.683 | 0.1183 |
| ACC | Postmenstrual age | vs. | GSH_ConcCr | -0.272 | -1.059 | 0.3074 |
| ACC | Postmenstrual age | vs. | GABA_ConcIU_TissCorr | 0.046 | 0.173 | 0.8653 |
| ACC | Postmenstrual age | vs. | Glx_ConcIU_TissCorr | -0.370 | -1.378 | 0.1935 |
| ACC | Postmenstrual age | vs. | GSH_ConcIU_TissCorr | -0.304 | -1.194 | 0.2522 |
| tha | Postmenstrual age | vs. | GABA_ConcCr | -0.295 | -1.069 | 0.3060 |
| tha | Postmenstrual age | vs. | Glx_ConcCr | -0.325 | -1.191 | 0.2567 |
| tha | Postmenstrual age | vs. | GSH_ConcCr | -0.206 | -0.697 | 0.5003 |
| tha | Postmenstrual age | vs. | GABA_ConcIU_TissCorr | -0.048 | -0.165 | 0.8715 |
| tha | Postmenstrual age | vs. | Glx_ConcIU_TissCorr | 0.003 | 0.010 | 0.9918 |
| tha | Postmenstrual age | vs. | GSH_ConcIU_TissCorr | -0.076 | -0.253 | 0.8050 |
